# Augmentation of Solid Tumor Immunotherapy With IL‐12

**DOI:** 10.1002/jgm.70000

**Published:** 2024-12-01

**Authors:** Christian Geils, Katie L. Kathrein

**Affiliations:** ^1^ Department of Biological Sciences University of South Carolina Columbia South Carolina USA

**Keywords:** gene delivery, gene therapy, tumor biology, tumor immunology, tumor targeting

## Abstract

*Immunotherapy* describes a class of therapies in which the immune system is manipulated for therapeutic benefit. These treatments include immune checkpoint inhibitors, adoptive cell therapy, and vaccines. For many hematological malignancies, immunotherapy has emerged as an essential treatment component. However, this success has yet to be replicated for solid tumors, which develop advanced physical and molecular mechanisms for suppressing and evading immune destruction. Nevertheless, cytokine immunotherapy presents a potential remedy to these barriers by delivering a proinflammatory immune signal to the tumor and thereby transforming it from immunologically “cold” to “hot.” Interleukin‐12 (IL‐12), one of the most potent proinflammatory cytokines, was initially investigated for this purpose. However, initial murine and human studies in which IL‐12 was administered systemically resulted in dangerous immunotoxicity associated with off‐target immune activation. As a result, recent studies have employed advanced cell and molecular engineering approaches to reduce IL‐12 toxicity while increasing or maintaining its efficacy such that its effective doses can be tolerated in humans. This review highlights such developments and identifies promising future directions.

## Graphical abstract

In this review, different integrations of the highly pro‐inflammatory cytokine interleukin‐12 (IL‐12) with existing treatments for solid tumors are highlighted. Promising directions in adoptive cell therapy, viral vectors, and protein engineering are identified and major challenges including a lack of translation of preclinical results to human trials, severe off‐target immunotoxicity, and the selection of complementary treatment are addressed. Finally, the proportions of different immune cell types in solid tumors are analyzed to identify tumors for which IL‐12 has the greatest potential.

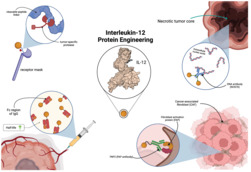

**Abbreviations**
αPD1: anti PD‐1 antibody
Ad‐RTS‐IL‐12: adenovirus with RTS‐mediated IL‐12 expression
ACT: adoptive cell therapy
ALL: acute lymphoid leukemia
APC: antigen‐presenting cells
BBB: blood‐brain barrier
CAF: cancer‐associated fibroblasts
CAR: chimeric antigen receptor
CD: cluster of differentiation
CTLA‐4: cytotoxic T lymphocyte antigen 4
EpCAM: epithelial cell adhesion molecule
FAP: fibroblast activation protein
Fc: crystallizable fragment
GBM: glioblastoma multiforme
hFAP: human FAP
hPBMC: human peripheral blood mononuclear cells
HSV‐1: Herpes Simplex Virus 1
huBC1: huBC1‐IL12
ICI: immune checkpoint inhibitors
IFP: interstitial fluid pressure
IgG: immunoglobulin class G
IL‐12: interleukin‐12
KIRC: kidney renal clear cell carcinoma
KIRP: kidney renal papillary cell carcinoma
mbIL‐12: membrane‐bound IL‐12
MHC: major histocompatibility complex
MMPs: matrix metalloproteinases
NFAT: nuclear factor of activated T cell
OR: objective responses
OV: oncolytic vectors
PD‐1: programmed cell death protein 1
PD‐L1: Programmed Cell Death Ligand 1
RTS: RheoSwitch Therapeutic System®
SKCM: skin cutaneous melanoma
SV: Sindbis virus
TAA: tumor‐associated antigens
TCT‐T: TCRs specific to tumor antigens
TIL: tumor‐infiltrating lymphocyte
TME: tumor microenvironment
Tregs: regulatory T cells
UCEC: uterine corpus cell carcinoma
UC: urothelial carcinoma
VSV: vesicular stomatitis virus.

## 1 Background

The role of the immune system in cancer has been extensively studied in recent years. This has unveiled both an antagonistic and cooperative relationship between tumors and different aspects of the immune system, which shapes tumor development. Of particular interest are solid tumors, whose immobility permits the development of complex physical and molecular mechanisms for exploiting and evading the immune system. Immune antagonism toward tumors is achieved through recognition and destruction of tumor cells by the immune system in a similar manner to extrinsic pathogens through presentation of abnormal antigens known as “tumor‐associated antigens” (TAAs) [1]. However, the ability of T cells to recognize TAAs can be suppressed by tumors directly through upregulation of inhibitory immune checkpoint molecules such as Programmed Cell Death Ligand 1 (PD‐L1), which suppress autoimmune responses in normal cells [2]. Furthermore, solid tumors can promote an immunosuppressive tumor microenvironment (TME) composed of tumor cells, immune cells, and secretory factors. Recruitment of immunosuppressive cells such as regulatory T cells (Tregs) and M2 macrophages suppress the cytotoxic functions of CD8+ T cells that would otherwise initiate an antitumor response to help promote an immunosuppress [3, 4, 5]. Lastly, the compression of tortuous, aberrantly grown blood vessels by rapidly proliferating tumor tissue coupled with inadequate lymphatic drainage results in a high interstitial fluid pressure (IFP) [6]. Increased IFP consequently acts as an additional physical barrier to cytotoxic lymphocyte entry into the tumor space and correlates with poor prognoses. This resulting collection of immunosuppressive features play a central role in the ability of the tumor microenvironment (TME) to promote tumor growth by facilitating a broad escape from antitumor immune responses.

Several major classes of immunotherapy have emerged to address these immunosuppressive characteristics. Immune checkpoint inhibitors (ICI) are monoclonal antibodies that block the action of molecules such as programmed cell death protein 1 (PD‐1) and cytotoxic T lymphocyte antigen 4 (CTLA‐4), which are expressed on the surface of T cells and are targeted by tumors to negatively regulate antitumor immune responses [7]. ICIs such as ipilimumab (anti‐CTLA‐4) and pembrolizumab (anti‐PD‐1) have both demonstrated success in advanced metastatic melanoma and non–small‐cell lung cancer, respectively [8, 9]. Another important class of immunotherapies, adoptive cell therapy (ACT), promises to target tumor cells for destruction by directly engineering patients' lymphocytes to have affinity for TAAs. Prominent examples of ACT include chimeric antigen receptor (CAR) T cell therapy and tumor‐infiltrating lymphocyte (TIL) therapy.

One major limitation of current immunotherapy approaches in solid tumors is that they rely on immune cell invasion into the tumor space. ICI therapy enhances T cell antitumor activity; however, this effect cannot materialize in solid tumors without sufficient populations of intratumoral T cells. ACT therapy shares this limitation, as the treatment is derived from a patient's own lymphocytes. It is not surprising, then, that immunotherapies targeting solid tumors have had limited success compared with hematological malignancies due to the immunosuppressive characteristics of the TME. Indeed, ICI therapy in solid tumors is characterized by high variation in responses with a large proportion of patients exhibiting no response [10]. ACT has faced similar challenges, with a general failure to translate its unprecedented successes in treating hematologic malignancies to solid tumors [11].

The solution to this broad limitation in solid tumor immunotherapy may lie in treatments that reshape the TME from immunologically “cold” to “hot.” Cytokine immunotherapy achieves this goal by leveraging the natural ability of cytokines to improve immune cell recruitment to the tumor space, decrease immune cell exhaustion phenotypes, promote tumor antigen expression, and prime immune cells for increased activation leading to an enhanced antitumor response [12].

**FIGURE 1:**
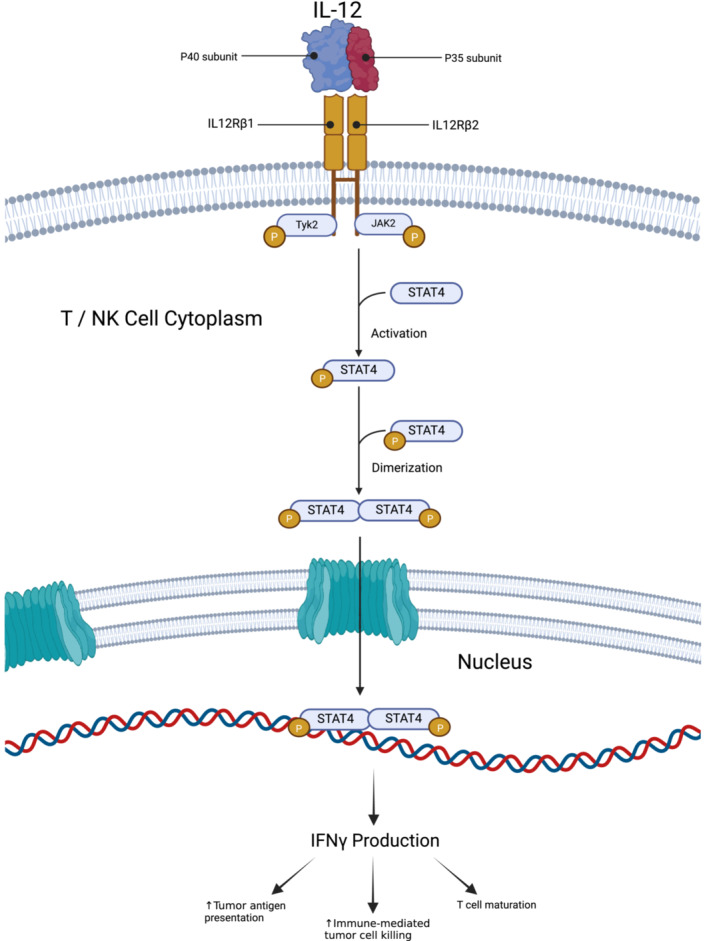
Interleukin‐12 (IL‐12) mechanism of action [13]. IL‐12 is composed of p40 and p53 subunits, which interact with the β1 and β2 subunits of the IL‐12 receptor, respectively. Binding to the receptor complex triggers phosphorylation of Janus Kinase (JAK) 2 and subsequent activation of STAT4. Dimerization of phospho‐STAT4 and translocation into the nucleus results in transcriptional changes, of which the primary contributor to IL‐12 potency is interferon gamma (IFNγ). IFNγ acts on multiple systems to increase tumor‐associated antigen (TAA) presentation, promote immune‐mediated tumor cell killing, and induce T cell maturation. Created using BioRender.

Interleukin‐12 (IL‐12) is one of the most potent proinflammatory cytokines (Figure 1). Specifically, it activates both cytotoxic CD8 T cells and NK cells, signaling for their expansion and maturation [14, 15]. IL‐12 acts by binding with its receptor subunits IL‐12Rβ1 and IL‐12Rβ2 and downstream activation of STAT3 and STAT4, which promote expression of the highly proinflammatory interferon gamma (IFNγ) [13]. IL‐12 was consequently an early target for solid tumor immunotherapy. The initial preclinical studies with intravenous murine IL‐12 demonstrated an ability to remodel the TME and facilitate a CD8+ T cell–dependent antitumor immune response [16]. However, murine models also revealed extensive off‐target immunotoxicity associated with IFNγ generation and IL‐12 binding outside the tumor space [17]. This limitation was significantly exacerbated in subsequent human studies with the first phase I and II trials resulting in severe toxicity, multiple hospital admissions, and three treatment‐related patient deaths [18, 19]. These disappointing initial findings resulted in a decline in IL‐12 cytokine related immunotherapy studies. However, recent years have seen a rapid proliferation of novel ICI, ACT, and other immunotherapy treatments whose efficacy depends largely on the activation of autologous T and NK cells toward tumors. This, coupled with advancements in cellular and protein engineering, has reengaged academic and industry interest in IL‐12 immunotherapy as a remedy to the limitations imposed by an immunosuppressive TME. Namely, new studies attempt to employ IL‐12 for solid tumor immunotherapy as either a combination or in a modified form to reduce its toxicity. This review highlights recent developments in IL‐12 immunotherapy while suggesting promising future directions.

## 2 Protein Engineering

Systemic administration of natural cytokines as antitumor agents has long been known to cause unfavorable toxicity due to their complex pleiotropic effects, which become difficult to control or predict when administered systemically in large amounts [20]. Thus, protein engineering methods are aimed at generating artificial cytokine constructs with reduced toxicity and greater specificity for the TME. Fusion of cytokines to antibodies targeting antigens expressed in the TME, known broadly as Immunocytokines, is a promising emerging strategy [21]. These fusion proteins aim to target tumor cells and the TME with greater specificity than their proinflammatory cytokine payloads alone, which include IL‐2, tumor necrosis factor (TNF), and IL‐12.

**FIGURE 2:**
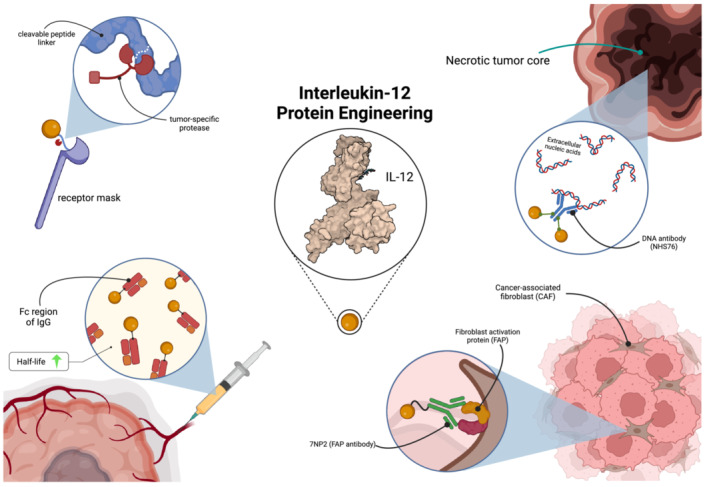
Protein engineering methods to augment IL‐12 immunotherapy. Various protein engineering methods have been applied to IL‐12 immunotherapy, with the goal of increasing specificity for the tumor while decreasing off‐target toxicity. Fusion to the β1 subunit of the IL‐12 receptor with a linker composed of a substrate for a tumor‐specific protease allows for IL‐12 release upon entering the tumor space [22]. An immunocytokine constructed from two IL‐12 moieties and a NHS76, an antibody to nucleic acids, directs IL‐12 to the necrotic tumor core [23]. IL12 joined to 7NP2 exploits the overabundance of cancer‐associated fibroblasts in solid tumors by targeting fibroblast activation protein (FAP) [24]. Lastly, fusion to a crystallizable fragment (Fc) of immunoglobulin G (IgG) increases serum half‐life, thus permitting less frequent dosing [25]. Created using BioRender.

The most well‐studied IL‐12 immunocytokine is comprised of NHS76 fused to two IL‐12 heterodimers, termed NHS‐IL12 (Figure 2). NHS76 is an engineered human monoclonal immunoglobulin G1 antibody that targets necrotic tumor tissues by binding extracellular nucleic acids [26]. Because solid tumors are often characterized by a necrotic core due to poor vascularization and nutrient deficiency, NHS76 was identified as a useful tool in directing IL‐12 to the tumor center [27]. Thus, the combination was expected to exhibit reduced toxicity compared with naked IL‐12. In mouse and *cynomolgus* monkey tumor model experiments, NHS‐IL12 exhibited promising antitumor effects when administered intravenously [23]. However, results from human trials were less successful (Table 1). In the first phase Ib human trial of NHS‐IL12, administered subcutaneously and combined with the anti‐PD‐L1 monoclonal antibody avelumab, only two of 32 patients with advanced solid tumors achieved objective responses (ORs) at data cut‐off [28]. In the dose expansion portion, evaluated on 16 patients with advanced urothelial carcinoma (UC), no tumor achieved ORs and, having fewer than three ORs, the treatment failed to meet the criterion for progression to stage 2. Furthermore, the results from dose‐expansion were inferior to prior studies employing avelumab monotherapy in patients with advanced UC, thus failing to support the hypothesis of a synergistic relationship between NHS‐IL‐12 and anti PD‐L1 immunotherapy [30]. It is not clear, with the small cohort of the dose expansion part, to what extent the severity of UC was replicated between the NHS‐IL12‐avelumab and avelumab monotherapy studies, and whether direct comparison is appropriate. Nevertheless, this underperformance may be explained in that NHS‐IL12 administration was followed by an increase in expression of PD‐L1 inside and outside the tumor space, acting as a pharmacological sink to avelumab. This mechanism suggests that the ability of IL‐12 constructs to augment ICI therapy may be restricted when both are administered systemically. Additionally, the ability of IL‐12 to exacerbate immune exhaustion by facilitating production of IFNγ, which is known to increase PD‐L1 expression, should be considered a major limitation to IL‐12 immunotherapy [31].

**TABLE 1:** Treatment outcomes for IL‐12 protein engineering human trials.

Treatment	Trial outcome	References
NHS‐IL12 + Avelumab (anti‐PD‐L1)	Failure: 2 ORs/32 subjects	[28]
huBC1‐IL12	Failure: 1 partial response/13 subjects	[29]

Constructed from a human monoclonal antibody targeting fibronectin joined to IL‐12, the development of huBC1‐IL12 (huBC1) suffered a similar fate to NHS‐IL12. huBC1 targets the cryptic portion of the oncofetal B‐FN isoform of fibronectin, which is a major component of the extracellular matrices of fetal and tumor tissue but not in healthy adult tissue [32]. In preclinical human tumor models and in xenogeneic human tumor murine models, huBC1‐IL12 demonstrated promising antitumor effects [33]. In a phase I human trial evaluating intravenous huBC1‐IL12 in 13 patients with malignant melanoma or renal cell carcinoma, only one partial response was observed [29]. Although the treatment was generally well tolerated and met the efficacy criteria for future trials, no such trials have been conducted.

A promising alternative target for immunocytokine therapy is fibroblast activation protein (FAP). FAP is highly expressed by cancer‐associated fibroblasts (CAFs) and is used as a universal marker for these cells [34]. FAP is known to play a role in angiogenesis, extracellular matrix remodeling, and cell signaling and is primarily associated with wound healing in normal tissues [35]. The role of FAP in cancer is controversial, with studies demonstrating both positive and negative prognostic correlations [36]. Nevertheless, the overexpression of FAP and thus the presence of large quantities of CAFs (20%–40% of total tumor mass) in 28 common tumors including breast, colorectal, pancreatic, and lung suggests that treatments targeting FAP may have broad applications for solid tumors [37]. Consequently, antibodies against FAP have emerged as an important component of antitumor therapies. Using the 7NP2 antibody to human FAP (hFAP), two variants of a novel IL12‐7NP2 fusion protein were generated (Figure 2), containing either human or murine IL‐12 [24]. In mouse models bearing either SKRC52 renal cell carcinoma or CT26 colon carcinoma expressing hFAP, weekly intravenous IL12‐7NP2 injections achieved complete responses in three out of six and two out of five mice, respectively. In CT26 models, subsequent combination with anti PD‐1 antibody (αPD1) achieved complete responses in all 3 animals compared with 0 in αPD1 monotherapy. The human IL12‐7NP2 demonstrated induction of IFNγ release in NK‐92 and human peripheral blood mononuclear cells (hPBMCs) and, in the same study, was tolerated in *Cynomolgus* monkeys up to 0.2 mg/kg, indicating IL12‐7NP2 should be evaluated in human trials [24].

In addition to immunocytokines, other cytokine fusion proteins are being explored, in part with the goal to increase the therapeutic window of IL‐12 by altering its half‐life, specifically in the TME [20]. With their short duration of exposure, conventional cytokine therapies require repeated administration, often daily, to achieve sufficient tumor uptake. The result of frequent IL‐12 dosing is a marked desensitization, with treatment cycles showing significant attenuation in IFNγ induction after two or three administrations [18]. One of the most common modifications made to address this challenge is the fusion of a crystallizable fragment (Fc) region of the immunoglobulin class G (IgG) antibody (Figure 2), which improves half‐life by increasing the molecular weight of the resulting peptide and through various receptor interactions [38]. Introduction of a D265A mutation prevents FcγR binding and complement activation, and thus the immunologic effects of the fusion protein are similar to the cytokine alone [39]. The Fc/IL‐2 fusion protein exhibits a serum half‐life roughly three times that of wild‐type IL‐12, but this did little to augment its efficacy against B16F10 melanoma mouse models [40]. More recently, a similar fusion protein was generated with IL‐12 as its payload, termed mDF6006. The IL‐12‐Fc substantially outperformed recombinant mouse IL‐12 (rmIL‐12) when administered intravenously to CT26 colon carcinoma mouse models, achieving complete tumor regression in all 10 animals compared with only one for rmIL‐12 [25]. By prolonging its serum half‐life from 6 to 30 h, dosing was reduced to weekly and still facilitated stable and prolonged accumulation of IFNγ. Furthermore, the fusion protein was effective against large (~815 mm^3^) CT26 tumor–bearing mice, also yielding 100% complete responses without significant differences in toxicity compared with rmIL‐12. These data are surprising and promising, considering large tumors are known to harbor microenvironments with greater populations of immunosuppressive Tregs, M2 macrophages, and myeloid‐derived suppressor cells, and thus generally respond poorly to immunotherapies compared with smaller tumors [41].

To increase the specificity of IL‐12 for the TME, IL‐12 was fused to a tumor‐protease‐cleavable peptide linker at the receptor binding site [22]. The resulting construct M‐L_6_‐IL‐12 consisted of IL‐12 fused to an IL‐12 receptor β1 (IL‐12Rβ1) via a substrate of matrix metalloproteinases (MMPs), which are known to be overexpressed in the TME as a mediator of extracellular matrix digestion and subsequent tumor expansion (Figure 2) [42]. Because IL‐12 has far greater affinity for the full receptor complex with both β1 and β2 subunits rather than just β2, the fused IL‐12Rβ1acts as a mask which inhibits cytotoxic activity until its cleavage by proteases in the TME, thus conferring on the complex preferential activity toward tumors [43]. In B16F10 and EMT6 melanoma mouse models, intravenous M‐L_6_‐IL‐12 and IL‐12 exhibited similar TME remodeling and antitumor effects, though with the former having no systemic immune‐related adverse events. Furthermore, M‐L_6_‐IL‐12 potentiated PD‐1 checkpoint blockade by increasing CD8+ T cell infiltration into the tumor space. These data suggest that augmentation of IL‐12 with peptide masks that are cleaved by proteases overexpressed in the TME may be a promising means of reducing systemic IL‐12 toxicity. The choice of linker could potentially by customized to the features of each patient's TME, thus giving the cytokine greater tumor specificity.

Based on findings from existing protein engineering studies employing IL‐12, certain approaches have the potential to augment IL‐12 such that its specificity for the TME is improved and its toxicity is reduced. However, a major limitation to systemically administered fusion proteins suggested by Kin‐Ming Lo et al. is that the high interstitial pressure of solid tumors may make the penetration of large peptides into the tumor space prohibitive. Future studies could attempt to address this limitation by exploring new fusion moieties of varying sizes and targets.

## 3 Adoptive Cell Therapy

In normal tissue, T cells perform surveillance of major histocompatibility complex (MHC) class I receptor presentation of antigens, to identify self‐ and non‐self‐peptides, where the latter category includes any peptides containing abnormal protein motifs. As a result, T cells are crucial for the detection of cancerous tissue, yet their activity is diminished due to the immunosuppressive TME of solid tumors. Thus, cancer cells may avoid detection as non‐self‐cells [44]. ACT describes a class of therapies in which immune cells are extracted from a patient, modified ex vivo, and reintroduced, with the goal of targeting and destroying tumor tissue [45]. Three major types of ACT have emerged. Tumor‐infiltrating lymphocyte (TIL) therapy, the first ACT to demonstrate clinical benefit, involves the extraction of immune cells from the tumor space, amplification in stimulatory medium, and readministration [46, 47]. TILs are advantageous in that they can be extracted, selected for antitumor characteristics, and expanded in vitro without the need for costly genetic modification or the selection of a tumor antigen [48]. This approach is restricted, however, to resectable tumors with sufficient populations of intratumoral T cells.

In attempt to address this limitation, T cells isolated from peripheral blood have been modified to express T cell receptors (TCRs) that specifically recognize tumors through tumor‐specific antigen presentation by MCH class I receptors (TCR‐T). This mechanism generates a finely tuned TCR that has strong affinity for tumor specific antigens, thus targeting tumor cells for destruction [49]. Although this approach has achieved some success in several liquid and solid tumors [50, 51], it possesses an inherent limitation in that TCRs can only bind antigens presented on by MHC class I [52]. The lifespan of many tumors is therefore characterized by a “Darwinian” selection process of tumor cells by cytotoxic T cells toward an MHC‐I‐negative tumor phenotype, leading to immune evasion and subsequent resistance to TCR‐T cell therapies [53]. CAR‐T cell therapy overcomes this limitation by employing a synthetic non–MHC‐restricted receptor as its targeting mechanism [54]. When directed toward CD19, CAR‐T cell therapies have achieved unprecedented success against hematological malignancies including acute lymphoid leukemia (ALL) and non‐Hodgkin lymphoma [55, 56].

However, ACTs have struggled to achieve the same clinical results for solid tumors as they have for hematologic malignancies. TIL therapy generated promising results in patients with more immunogenic solid tumor types, primarily melanoma [57], but has had limited success with other solid tumors. Levels of antigen targets, including cluster of differentiation (CD) molecules, are retained after transformation to hematologic malignancies and the targeted effects on nonmalignant cells are more clinically manageable [58, 59]. However, in solid tumors, the selection of target antigen for TCR‐T and CAR‐T cell therapies is difficult due to the lack of a uniformly expressed TAA [60]. These limitations are compounded by the immunosuppressive TME, which prevents ACT invasion into the tumor space in a similar manner to their nonengineered counterparts. Although IL‐12 cannot resolve the challenge associated with selecting a target antigen, its ability to reprogram the TME may act as a tool to improve ACT perfusion into solid tumors [61]. Furthermore, the ability of IL‐12 to prolong T cell survival and signal for clonal expansion suggests that ACTs expressing or bound to IL‐12 may act as an autocrine signal for activation upon binding to their target antigen [62, 63].

**FIGURE 3:**
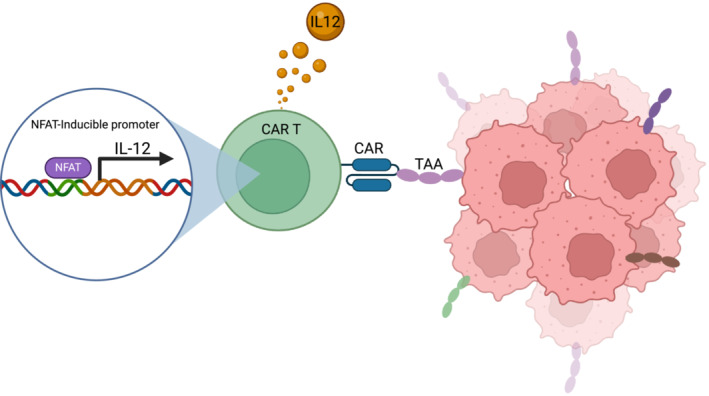
IL‐12 integrated into CAR T cell genome with NFAT‐inducible promoter. Using a viral vector, IL‐12 DNA joined with an NFAT‐inducible promoter can be inserted into the genome of CAR T cells. Because TCR/CAR recognition of its target antigen engages NFAT‐calcineurin signaling, this arrangement confers on the CAR T cells a tumor‐dependent ability to produce IL‐12 [65]. Created using BioRender.

There are several ways in which IL‐12 has been integrated into ACT. The first attempt to combine IL‐12 with ACT therapy via genome integration was done by genetically modifying TCR‐T cells to express IL‐12. This approach showed promising antitumor effects in lymphodepleted B16F10 melanoma mouse models when administered intravenously by tail vein, with only 10,000 modified T cells achieving similar antitumor effects to 1 million unmodified cells lacking IL‐12 expression at 25 days post‐infusion [64]. Concerns were raised over immunotoxicity as the transduced IL‐12 could be expressed continuously by the modified T cells, potentially triggering severe autoimmune responses outside the tumor space. As a result, CAR‐T cells with IL‐12 expression controlled by a nuclear factor of activated T cell (NFAT) promoter have been developed [65]. Because NFAT‐calcineurin signaling is an essential part of T cell activation [66], these inducible IL‐12 CAR‐T cells express IL‐12 preferentially in the tumor space upon recognition of their target antigen (Figure 3). With these cells exhibiting lower off‐target toxicity, future trials have continued to employ NFAT to regulate IL‐12 production.

Employing both NFAT‐IL12 and fine‐tuning CAR specificity for solid tumors has shown promising improvements [67]. In carcinomas, epithelial cell adhesion molecule (EpCAM) is expressed at levels far higher than those of normal epithelial cells. Fine‐tuning of CARs targeted to EpCAM to reduce CAR affinity toward EpCAM restricted their activity to tumor cells with high EpCAM expression. This loss of TAA affinity was compensated for by NFAT‐inducible IL‐12, which restored CAR activity to a level comparable to its non–affinity‐mutated parent. This combination reduces off‐target toxicity toward nonmalignant cells with normal EpCAM expression levels, to direct reactivity toward tumor cells with high expression. In thyroid carcinoma and gastric cancer mouse models, subcutaneous administration of inducible IL‐12 and affinity‐tuned CAR‐T (Y6V‐iIL12 CAR‐T) cells was significantly more effective compared with placebo or CAR‐T cells without IL‐12 in reducing tumor volume at 2–3 weeks post‐infusion. Furthermore, mice treated with Y6V‐iIL12 CAR‐T exhibited no systemic toxicity or body weight loss. These results suggest that combining both affinity‐tuning methods and inducible IL‐12 may allow for greater specificity without compromising antitumor activity for CAR‐T cells.

NFAT‐IL12 has additionally shown promise in combination with TIL therapy (Table 2). In 33 patients with metastatic melanoma treated with TILs transduced with NFAT‐IL12 via a viral vector, a promising 63% (10/16) achieved objective response at doses between 0.3–3 × 10^9^ cells. This result is despite the exclusion of IL‐2 to signal for TIL expansion as well as the fact that doses consisted of 2 to 3 orders of magnitude fewer total TIL cells [68]. Although this suggests that NFAT‐IL12 + TIL may reduce the total necessary dosage, the treatment was nonetheless accompanied by significant immunotoxicity with adverse events including severe fevers, febrile neutropenia, and grade 4 hepatotoxicity.

**TABLE 2:** Treatment outcomes for IL‐12 combinations with adoptive cell therapy in human trials.

Treatment	Trial outcome	References
TILs + NFAT‐IL12	Success: 10 ORs/16 subjects	[68]

IL12‐CAR‐T combinations using mRNA for IL‐12 expression has also been explored. This approach is advantageous compared with direct genomic integration due to its reduced manufacturing complexity; using electroporation to apply an electric pulse to cells to generate pores in the membrane through which genetic material can pass [69]. This bypasses the need for prior development of viral vectors or lipid nanovesicles. However, this also presents as a limitation due to the limited half‐life of the mRNA molecules that will result in translation of fewer total cytokines before degradation, whereas genomic integration allows for sustained and continuous IL‐12 release. These properties imply that mRNA‐based IL12‐CAR‐T therapies may be useful in cases where sustained cytokine release is unfavorable. One such tumor is glioblastoma, for which the development of cytokine‐CAR‐T cell therapies has been restricted, in part, due to concerns regarding neurotoxicity and cerebral oedema‐related deaths observed in prior CAR‐T cell trials [70]. Electroporation was used to insert both IL‐12 and IFNα2, another proinflammatory cytokine, into a multitargeting CAR‐T cell [71]. Against glioma mouse models, CAR‐T cells expressing IL‐12 and IFNα2 achieved superior overall survival at 60 days post‐infusion compared with cytokine monotherapy or standard CAR‐T cells when administered both locally and systemically. Although it is promising that no signs of toxicity were observed, it should be expected, as with all IL‐12 therapies, that human trials will demonstrate significantly greater toxicity. Nevertheless, transiently expressed IL‐12 achieves greater tolerability than when its expression is sustained, as in the case of direct genome integration.

The use of IL‐12 mRNA has also been investigated in TCR‐T cells. In B16F10 melanoma mouse models, intracavitary injection of ovalbumin‐targeting TCR‐T cells electroporated with IL‐12 mRNA achieved nearly 100% survival compared with less than 25% for all other treatments including intravenous injection, TCR‐T cells with irrelevant (non‐IL12) mRNAs, and control [72]. This result is significant because intracavitary administration of cytokine‐engineered ACTs to treat peritoneal malignancies has been restricted by concerns over toxicity despite the well‐documented superiority of locoregional immunotherapy administration for solid tumors [73, 74]. By exploiting the transient nature of mRNA, Di Trani et al. demonstrate how this limitation might be addressed.

ACT‐IL12 combinations have also been attempted by anchoring IL‐12 constructs to the membrane of CAR‐T cells. The addition of an antigen‐dependent membrane‐bound IL‐12 (mbIL12) to the surface of CAR‐T resulted in an autocrine signaling mechanism that improved antitumor efficacy in vitro and in vivo [75]. When administered locoregionally to ovarian cancer mouse models, CAR/mbIL12 T cells targeting the TAG72 antigen, reduction in tumor volume was superior to those treated with TAG72‐CAR‐T cells lacking IL‐12. Similarly to ACT treatments which require IL‐12 expression from genetic material, this combination demonstrated an ability to improve tumor penetration, T cell activation, and subsequent tumor killing. However, this approach, whereby IL‐12 is directly bound to T cells rather than expressed post‐infusion, is a potential remedy to the still‐present challenge in adequately controlling IL‐12 expression through promoters to reduce toxicity. This is because membrane‐bound IL‐12 is unable to diffuse to other tissues as is the case when it is produced by the cell and deposited into serum.

Membrane‐binding implementations have also been attempted by employing nanovesicles anchored to the cell surface. One such study conjugated a nanochaperone (INS) containing IL‐12 by chemical attachment with dibenzocyclooctyl (DBCO) groups [76]. By increasing redox activity at the cell surface upon antigen binding and T cell activation, experimental data showed that the IL‐12‐containing nanochaperone could be disseminated through disruption of its DBCO attachment. In solid B‐cell‐derived tumor mouse models, intravenous treatment with INS‐CAR‐T cells resulted in little to no tumor growth at day 31 compared with significant increases in tumor volume for CAR‐T cells alone or with free IL‐12. The use of nanovesicles as a means of anchoring antigen‐dependent IL‐12 to CAR‐T cells may help to further attenuate toxicity.

Certain challenges faced in applying ACT to solid tumors are yet to be resolved, including selection of tumor antigens from a vast library of heterogeneously expressed surface markers, rapid clearance from the body, and a time‐consuming and laborious manufacturing process. Although cytokine immunotherapies, including IL‐12, cannot fully address these limitations, they nonetheless have the potential to improve ACT penetration into solid tumors and enhance activation and tumor killing. However, the risk of off‐target toxicity already associated with many ACTs makes the integration with further immunostimulatory particles challenging and often unfavorable. Therefore, advanced cell and protein engineering techniques are required to fully exploit the benefit of ACT for solid tumors as has been observed in hematologic cancers. Finally, the inability to predict toxicology findings in clinical trials based on mouse trials warrants caution and suggests that other animal models that better approximate human physiology would be useful in cytokine‐based immunotherapies.

## 4 Viral Vectors

Tumor cells may be targeted for destruction by gene therapy, whereby exogenous, antitumor genes are delivered in the form of DNA or RNA and subsequently expressed to achieve an antitumor effect. Viral vectors constitute approximately 70% of current gene therapy trials due to their high gene transduction efficiency compared with nonviral vectors [77, 78]. Viral vector engineering can reduce pathogenicity, increase proliferation in tumors while decreasing in normal tissue, and evade the host immune response [78]. Vectors that have been engineered to be replication‐competent in tumor cells, or oncolytic vectors (OVs), have emerged as effective dual‐action treatments by inducing lysis and cell death through both proliferation and antitumor gene expression [79].

**FIGURE 4:**
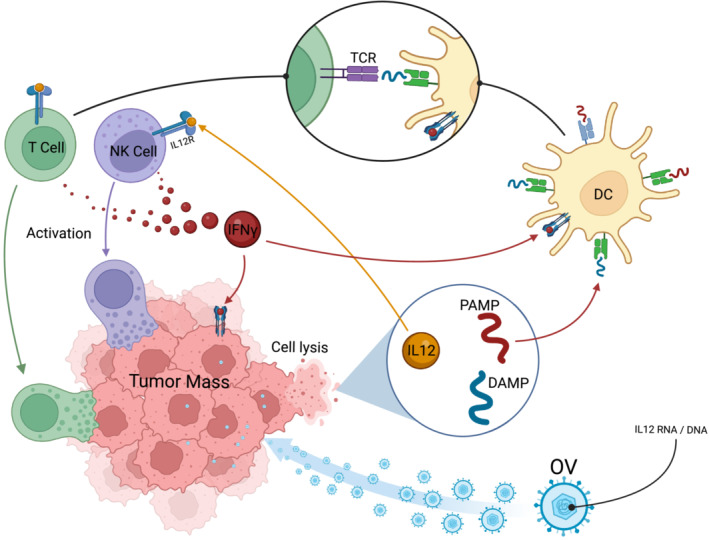
Interleukin‐12‐expressing oncolytic viruses (OVs) act on multiple systems to exert antitumor activity [80]. OVs exert antitumor activity by lysing tumor cells, thus releasing their associated antigens. These antigens can then be taken up by APCs such as dendritic cells (DCs) and then presented to the adaptive immune system to activate it against tumor cells. The cell types involved in this response primarily include cytotoxic T cells and NK cells. Furthermore, OVs can integrate IL‐12 directly into tumor genomes, resulting in IL‐12 expression by solid tumors and subsequent activation of T and NK cells. Downstream release of IFNγ results in expression of the chemokine IP‐10 by DCs, which interferes with tumor angiogenesis. Lastly, tumor necrosis factor alpha (TNF‐α) released by either activated DCs or cytotoxic lymphocytes induces tumor cell death. Created using BioRender.

Viral vectors encoding IL‐12 offer the ability to integrate and induce expression of the cytokine directly in the TME, thus simultaneously improving tumor penetration and stimulating an antitumor response by recruiting additional immune cells (Figure 4). Additionally, destruction of tumor cells by OVs results in the release of a debris field consisting of pathogen‐associated molecular patterns and damage‐associated molecular patterns to be taken up and presented by antigen‐presenting cells (APCs), contributing to an additional downstream recruitment of cytotoxic lymphocytes [81]. Together, these properties suggest a possible synergistic relationship between OVs and IL‐12.

Initial approaches integrated IL‐12 into retroviral or adenovirus platforms. However, murine models demonstrated high rates of adverse events associated with a post‐treatment spike in IFNγ coupled with a risk of off‐target integration into the host genome, which became significant limitations [82]. Nevertheless, an adenovirus encoding IL‐12 was tested on advanced digestive tumors in human subjects by intratumoral injection (Table 3) [83]. Subjects exhibited mild antitumor effects with 38% having tumor progression, 48% with stable disease, and one patient achieving partial remission. These results suggest a need for novel IL‐12 vectors with superior antitumor effects at tolerated doses.

**TABLE 3:** Treatment outcomes for IL‐12 combinations with viral vectors in human trials.

Treatment	Trial outcome	References
Adenovirus encoding IL‐12	Moderate success: 1 partial response + 6 stable disease/21 subjects	[83]
HSV‐1 expressing IL‐12	Moderate success: 9.38 months median survival vs. ~7 months in previous recurrent glioma trials	[84]
Recombinant adenovirus with regulatable IL‐12 expression via oral veledimex	Success: 12.7 months median survival vs. ~7 months in previous recurrent glioma trials	[85]

Alphaviruses such as Semliki Forest virus, Sindbis virus, and Venezuelan equine encephalitis virus previously demonstrated success as antitumor agents when engineered to induce overexpression of TAAs such as human papilloma virus type 16 E7 or antitumor genes such as endostatin [86, 87]. By delivering self‐replicating RNA to tumor cells, alphaviruses bypass the need to transfer genetic material directly into cell nuclei and integrate into the host genome as with DNA‐based vectors such as adenoviruses [88]. Furthermore, expression of corresponding peptides is superior to mRNA delivery, for which self‐replication is impossible. Alphaviruses have previously been applied as vectors for immunostimulatory agents, with those expressing IL‐12 being the most effective [88]. One of the earliest attempts used a Semliki Forest virus as a mediator of IL‐12 gene therapy, administered intratumorally via cannula, to a RG2 rat glioma model [89]. Results from this study were promising, with low and high‐dose achieving 70 and 87% reduction in tumor volume, respectively, though with a risk of lethality at high doses.

This limitation necessitated the development of novel alphavirus vectors with superior safety profiles. Sindbis virus (SV) was recently shown to be effective against Mouse Ovarian Surface Epithelial Cell Line models when encoding IL‐12 and an OX40 antibody and administered systemically [90]. Activation of T cell–expressed OX40 by anti‐OX40 in the TME promotes cytotoxic function and suppresses the action of Tregs, thus enhancing the anti‐tumor immune response. Results showed that SV expressing IL‐12 and anti‐OX40 resulted in greater survival and suppression of tumor growth than for an empty vector or one encoding IL‐12 or anti‐OX40 alone. Due to its long half‐life in the blood and resistance to neutralization by serum antibodies, SV can be administered systemically rather than intratumorally with little impact on its efficacy. This feature of SV could prove useful for patients where intratumoral administration is prohibitive, such as those where multiple lesions are present or for neurological malignancies where direct injection risks damage to surrounding tissue. Human trials are still needed to evaluate the safety and efficacy of alphavirus‐IL‐12 combinations.

Herpes Simplex Virus 1 (HSV‐1) has been evaluated using a similar, but slightly modified strategy: using expression of IL‐12 and OX40L to improve TIL therapy. As TIL therapy has previously been limited by insufficient activation and persistence in solid tumors, this vector was seen as a potential remedy. With this strategy, transformed tumor cells express and present OX40L to function as artificial antigen presenting cells (aAPCs) to trigger T cell activation with IL‐12 to transformation the TME and improve the efficacy of combination TIL therapy [91]. In an in vitro tumor model and two oral cancer patient‐derived xenograft (PDX) mouse models, the intratumor OV‐mOX40L/IL12 and intravenous TIL combination was shown to be more effective in initiating tumor shrinkage than control or any of the components alone. OV was not detected in peripheral tissues, suggesting a favorable safety profile. These results suggest the potential for viral vectors incorporating IL‐12 to address the limitations of combination ACT.

Viral vector approaches incorporating HSV or similar vectors may be uniquely effective in treating brain tumors due to their ability to penetrate the blood‐brain barrier (BBB), a major limitation observed in many treatments for neurologic pathologies. Moreover, their limited ability to replicate and spread through the TME may be augmented by combination with IL‐12 [92]. Indeed, neuroblastoma murine models showed that HSV‐1 engineered to express IL‐12 (HSV M002) was more effective than its nonengineered counterpart when administered intratumorally (HSV R3659) in improving post‐tumor‐induction survival [93]. In a similar study, 3D bioprinting was used to replicate the physical properties of human tumors in order test M002 against three human neuroblastoma PDXs [94]. This approach may more reliably predict the outcome of future human trials than mouse models. To this point, one phase I trial has been conducted testing a second‐generation HSV‐1 engineered to express IL‐12 (HSV‐1 M032) [84]. In patients with recurrent or progressive malignant glioma, an acceptable safety profile was observed for M032 administered via intratumoral catheter, with a median OS of 9.38 months (Table 3). Though not placebo‐controlled, this result is superior to previous recurrent glioma trials having a median OS of ~7 months [95]. With glioma being one of the deadliest solid tumors, and especially because it is characterized by one of the most intensely immunosuppressive TMEs, a combination of oncolytic viruses with IL‐12 proves to be a promising approach.

Engineered rhabdoviruses may also be useful as antitumor agents, with previous studies demonstrating their success as cancer vaccines due to their high immunogenicity and the precision with which their small genome can be manipulated [96, 97]. Of the rhabdovirus family, the vesicular stomatitis virus (VSV) is the most promising. The inability of VSV to invade and infect healthy cells with normal type‐I IFN expression confers specificity for the TME, where the type‐I IFN signaling axis is frequently deficient [98]. This suggests a potential to bypass off‐target IL‐12 toxicity. One study investigated this potential by constructing a recombinant VSVΔ51 expressing human IL‐12 [99]. Intratumoral treatment of a B16F10 melanoma mouse model showed prolonged survival for the recombinant vector compared with control and VSV alone. Furthermore, by deleting its M protein, which is responsible for VSV cytotoxicity toward healthy cells, VSVΔ51 was shown to have diminished cytotoxic effects against normal GM‐38 cells with or without IL‐12. Taken together, these findings suggest promising means by which VSV might be augmented to improve its safety and efficacy through incorporation with IL‐12.

Systemic IL‐12 toxicity remains a concern for these treatments, and mechanisms to address this are being considered. Selective control of IL‐12 expression after integration by a viral vector may be achieved through use of The RheoSwitch Therapeutic System® (RTS), which facilitates viral integration of a veledimex (VDX) – activated transgene. This system was shown to be effective in regulating IL‐12 expression when injected intratumorally [100]. The recombinant adenovirus with RTS‐mediated IL‐12 expression (Ad‐RTS‐IL‐12) allowed for precise control of intratumoral IL‐12 expression with oral VDX in monkey and mouse glioma models, with higher doses resulting in significantly greater percent survival compared with the current standards of care (bevacizumab and temozolamide) and control. Importantly, the treatment resulted in no severe adverse events and 95% of animals in the Ad‐RTS‐IL‐12 treatment groups were tumor‐free at study termination. In one of the few human trials of its kind, intratumoral Ad‐RTS‐IL‐12 expression with oral VDX was tested as a treatment for recurrent high‐grade glioma [85]. Results confirmed that hIL‐12 expression was tightly controlled by RTS and that VDX successfully crosses the BBB. Adverse events were mild to moderate, with promising evidence of TME remodeling and survival probability compared with historical controls (Table 3). These findings suggest that the toxicity of IL‐12, which previously limited its feasible dosage and thus efficacy in human trials, could be reduced by systems that allow for ligand‐controlled expression such as RTS.

By integrating IL‐12 DNA or RNA directly into tumor tissue, viral vectors may improve selectivity of IL‐12 treatments and thus increase the amount of IL‐12 that can safely be administered to a patient without severe off‐target immunotoxicity. Furthermore, the destruction of tumor cells by those vectors with oncolytic potential, thus releasing a debris field of TAAs to be recognized by cytotoxic lymphocytes recruited to the TME by IL‐12 may act as a synergistic mechanism. However, the risk of random integration into the host genome and frequent neutralization by innate and adaptive immune responses continue to pose limitations for many of these therapies.

## 5 The Tumor Microenvironment

**FIGURE 5:**
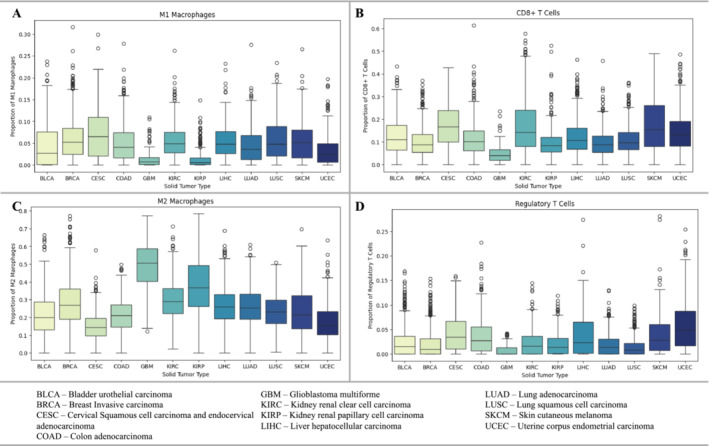
Immune cell proportions in the tumor microenvironment of common solid tumors. Proportions of immune cells in 10 most common solid tumor types based on >10,000 tumor samples. Immune fractions are calculated using CIBERSORT, which estimates cell type abundance using single‐cell RNA‐sequencing data [102]. (A) M1 Macrophages and (B) CD8+ T cells promote antitumor immunity, whereas (C) M2 macrophages and (D) Regulatory T cells suppress it.

The use of IL‐12 in solid tumor immunotherapy is predicated on two of its properties: the ability to remodel the immunosuppressive TME and activate immune cells toward an antitumor response. By characterizing the TME for different solid tumor types, the precise utility of IL‐12 might be elucidated. Interaction between IL‐12 and tumor‐associated macrophages has been observed to rapidly induce a transition from a tumor‐promoting anti‐inflammatory M2 phenotype (characterized by production of IL‐10, MCP‐1, and TGFβ), to an anti‐tumor proinflammatory M1 phenotype with increased production of TNF‐α, IL‐15, and IL‐18 [101]. This, in combination with the potent stimulatory action of IL‐12 toward CD8+ T cells, suggests that the proportions of immune cell populations in common solid tumor types may inform which cancer types would benefit from this therapy (Figure 5) [102]. As a result, these profiles suggest that kidney renal papillary cell carcinoma (KIRP) and uterine corpus cell carcinoma (UCEC) may be a possible target for treatment. Although glioblastoma multiforme (GBM), with low populations of M1 macrophages (Figure 5A), might benefit from IL‐12 exposure, the low populations of CD8+ T cells may exhibit a diminished antitumor response with IL‐12 treatment. Tumors with greater populations of CD8+ T cells such as skin cutaneous melanoma (SKCM) and kidney renal clear cell carcinoma (KIRC) may exhibit greater antitumor activity in response to IL‐12 (Figure 5B). Indeed, many of the best clinical and preclinical outcomes for IL‐12 immunotherapy have been observed in either advanced human melanoma or B16F10 melanoma mouse models.

The TME of GBM and KIRP also show comparatively large populations of immunosuppressive M2 macrophages (Figure 5C). As IL‐12 converts M2 macrophages to their antitumor M1 phenotype, it should be expected that this feature of solid tumors can be addressed [103].

Lastly, high proportions of Tregs can be observed in uterine corpus endometrial carcinoma and cervical squamous cell carcinoma (Figure 5D). Tregs are a subset of CD4+ T cells which induce immunosuppression through upregulation of the immune checkpoint CTLA‐4 and through the production of immunosuppressive cytokines including IL‐10, IL‐35, and TGFβ [104]. The ability of IL‐12 to reduce IL‐2 expression, which is essential for Treg survival and expansion, and to stimulate IFNγ‐dependent Treg cell cycle arrest suggests a potential corrective mechanism for tumors which exploit the immunosuppressive effects of Tregs [105].

## 6 Conclusions

Recent advancements in cell and protein engineering have revitalized scientific interest in cytokine immunotherapy for solid tumors, with IL‐12 being a chief benefactor. The initial limitations faced by IL‐12 have been addressed by integrating them into the genome of T cells as part of ACT, transduced using oncolytic viral vectors, and engineered to better target solid tumors and persist in the TME. Nevertheless, the severe immunotoxicity observed in early IL‐12 preclinical and clinical trials continues to limit safe dosages to ones with insufficient product to achieve acceptable antitumor efficacy. This is especially true in human trials, where results from murine models fail to predict the relationship between efficacy and toxicity. There are a number of possible hypotheses for why this might be the case. First, murine models with implanted tumors may not accurately represent the physical barriers posed by naturally occurring tumors, which can exist in multiple parts or integrate themselves deeply into healthy tissue. Another possible explanation is that human tumors are much smaller, as a percentage of total body weight and serum volume, than tumors used in mouse models. As an example, a 0.2‐cm^3^ oral squamous cell carcinoma in a mouse model with a body weight of 20 g used in one IL‐12 study would be the equivalent, based on volume‐to‐mass ratio, of a 12,000‐cm^3^ tumor with a diameter of ~28.4 cm for a 60‐kg man [106]. This size is significantly greater than those reported for the vast majority of human solid tumors, which typically average around 2–5 cm in diameter [107, 108]. Lastly, differences in responses to IL‐12 may be explained by differences in receptor expression, immune cell populations, and the quantity and behavior of downstream signaling components such as STAT4 and IFNγ. In order to advance IL‐12 immunotherapy, it is necessary to identify tumor models that better replicate the features of human tumors.

Additionally, the method of administration of IL‐12 may play a role in its efficacy. Administration either via cannula or by injection directly into the tumor space is preferable, with systemic IL‐12 necessarily incurring a far greater risk of off‐target tumor toxicity. However, this is prohibited in tumors for which their location prevents intratumoral administration or where multiple tumors are present. As a result, it is necessary to develop methods of administration, which address these limitations. One potential approach would be the use of multiple cannulas simultaneously for fragmented tumors. For tumors such as glioblastoma, novel surgical methods may be necessary to safely administer IL‐12 without damaging surrounding tissue.

IL‐12 represents an important potential tool to address a major limitation in current immunotherapies: the tumor microenvironment. Its powerful proinflammatory features enable it to reshape the TME to the benefit of the patient, though with a risk of toxicity. Future IL‐12 studies should continue to explore novel methods to reduce the toxicity of IL‐12 therapies such that its safe dosage rises to the level of its effective dosage, and should innovate on preclinical testing and administrative methods.

## Author Contributions

CG and KLK were responsible for conceptualization, data curation, manuscript preparation, and reviewing and editing. Both authors read and approved the final version of the manuscript submitted for publication.

## Ethics Statement

No animals or human subjects were used in the preparation of this review article. All analyzed datasets are publicly available and anonymized.

## Conflicts of Interest

The authors declare no conflicts of interest.

## Data Availability

All datasets used or analyzed during the current study are publicly available.

## References

[1] P. G. Coulie , B. J. Van den Eynde , P. van der Bruggen , and T. Boon , “Tumour Antigens Recognized by T Lymphocytes: At the Core of Cancer Immunotherapy,” Nature Reviews Cancer 14, no. 2 (2014): 135–146, 10.1038/nrc3670. 24457417

[2] Y. Han , D. Liu , and L. Li , “PD‐1/PD‐L1 Pathway: Current Researches in Cancer,” American Journal of Cancer Research 10, no. 3 (2020): 727–742, https://www.ncbi.nlm.nih.gov/pmc/articles/PMC7136921/. 32266087 PMC7136921

[3] J.‐H. Kim , B. S. Kim , and S.‐K. Lee , “Regulatory T Cells in Tumor Microenvironment and Approach for Anticancer Immunotherapy,” Immune Network 20, no. 1 (2020): e4, 10.4110/in.2020.20.e4. 32158592 PMC7049587

[4] Y. Xu , X. Wang , L. Liu , J. Wang , J. Wu , and C. Sun , “Role of Macrophages in Tumor Progression and Therapy (Review),” International Journal of Oncology 60, no. 5 (2022): 57, 10.3892/ijo.2022.5347. 35362544 PMC8997338

[5] N. M. Anderson and M. C. Simon , “The Tumor Microenvironment,” Current Biology: CB 30, no. 16 (2020): R921‐R925, 10.1016/j.cub.2020.06.081. 32810447 PMC8194051

[6] S. Ferretti , P. R. Allegrini , M. M. Becquet , and P. M. McSheehy , “Tumor Interstitial Fluid Pressure as an Early‐Response Marker for Anticancer Therapeutics,” Neoplasia (New York, N.Y.) 11, no. 9 (2009): 874–881, https://www.ncbi.nlm.nih.gov/pmc/articles/PMC2735799/. 19724681 10.1593/neo.09554 PMC2735799

[7] Y. Shiravand , F. Khodadadi , S. M. A. Kashani , et al., “Immune Checkpoint Inhibitors in Cancer Therapy,” Current Oncology 29, no. 5 (2022): 3044–3060, 10.3390/curroncol29050247. 35621637 PMC9139602

[8] M. Mansh , “Ipilimumab and Cancer Immunotherapy: A New Hope for Advanced Stage Melanoma,” Yale Journal of Biology and Medicine 84, no. 4 (2011): 381–389, https://www.ncbi.nlm.nih.gov/pmc/articles/PMC3238313/. 22180676 PMC3238313

[9] R. Martin , R.‐A. Delvys , A. G. Robinson , et al., “Pembrolizumab Versus Chemotherapy for PD‐L1–Positive Non–Small‐Cell Lung Cancer,” New England Journal of Medicine 375, no. 19 (2016): 1823‐1833, 10.1056/NEJMoa1606774. 27718847

[10] Q. Sun , Z. Hong , C. Zhang , L. Wang , Z. Han , and D. Ma , “Immune Checkpoint Therapy for Solid Tumours: Clinical Dilemmas and Future Trends,” Signal Transduction and Targeted Therapy 8, no. 1 (2023): 1–26, 10.1038/s41392-023-01522-4. 37635168 PMC10460796

[11] D. J. Olson and K. Odunsi , “Adoptive Cell Therapy for Nonhematologic Solid Tumors,” Journal of Clinical Oncology 41, no. 18 (2023): 3397–3407, 10.1200/JCO.22.01618. 37104722

[12] K. Song , “Current Development Status of Cytokines for Cancer Immunotherapy,” Biomolecules & Therapeutics 32, no. 1 (2024): 13–24, 10.4062/biomolther.2023.196. 38148550 PMC10762268

[13] C. T. Weaver , R. D. Hatton , P. R. Mangan , and L. E. Harrington , “IL‐17 Family Cytokines and the Expanding Diversity of Effector T Cell Lineages,” Annual Review of Immunology 25 (2007): 821–852, 10.1146/annurev.immunol.25.022106.141557. 17201677

[14] U. Gubler , A. O. Chua , D. S. Schoenhaut , et al., “Coexpression of Two Distinct Genes Is Required to Generate Secreted Bioactive Cytotoxic Lymphocyte Maturation Factor,” Proceedings of the National Academy of Sciences of the United States of America 88, no. 10 (1991): 4143–4147, https://www.ncbi.nlm.nih.gov/pmc/articles/PMC51614/. 1674604 10.1073/pnas.88.10.4143 PMC51614

[15] A. S. Stern , F. J. Podlaski , J. D. Hulmes , et al., “Purification to Homogeneity and Partial Characterization of Cytotoxic Lymphocyte Maturation Factor From Human B‐Lymphoblastoid Cells,” Proceedings of the National Academy of Sciences of the United States of America 87, no. 17 (1990): 6808–6812, https://www.ncbi.nlm.nih.gov/pmc/articles/PMC54627/. 2204066 10.1073/pnas.87.17.6808 PMC54627

[16] M. J. Brunda , L. Luistro , R. R. Warrier , et al., “Antitumor and Antimetastatic Activity of Interleukin 12 Against Murine Tumors,” Journal of Experimental Medicine 178, no. 4 (1993): 1223–1230, 10.1084/jem.178.4.1223. 8104230 PMC2191194

[17] M. K. Gately , R. R. Warrier , S. Honasoge , et al., “Administration of Recombinant IL‐12 to Normal Mice Enhances Cytolytic Lymphocyte Activity and Induces Production of IFN‐γ in Vivo,” International Immunology 6, no. 1 (1994): 157–167, 10.1093/intimm/6.1.157. 7908534

[18] M. B. Atkins , M. J. Robertson , M. Gordon , et al., “Phase I Evaluation of Intravenous Recombinant Human Interleukin 12 in Patients With Advanced Malignancies,” Clinical Cancer Research 3, no. 3 (1997): 409–417. 9815699

[19] J. P. Leonard , M. L. Sherman , G. L. Fisher , et al., “Effects of Single‐Dose Interleukin‐12 Exposure on Interleukin‐12–Associated Toxicity and Interferon‐γ Production,” Blood 90, no. 7 (1997): 2541–2548, 10.1182/blood.V90.7.2541. 9326219

[20] L. Santollani and K. D. Wittrup , “Spatiotemporally Programming Cytokine Immunotherapies Through Protein Engineering,” Immunological Reviews 320, no. 1 (2023): 10–28, 10.1111/imr.13234. 37409481

[21] A. Pabani and J. F. Gainor , “Facts and Hopes: Immunocytokines for Cancer Immunotherapy,” Clinical Cancer Research 29, no. 19 (2023): 3841–3849, 10.1158/1078-0432.CCR-22-1837. 37227449

[22] A. Mansurov , P. Hosseinchi , K. Chang , et al., “Masking the Immunotoxicity of Interleukin‐12 by Fusing It With a Domain of Its Receptor via a Tumour‐Protease‐Cleavable Linker,” Nature Biomedical Engineering 6, no. 7 (2022): 819–829, 10.1038/s41551-022-00888-0. PMC11155269 35534574

[23] J. Fallon , R. Tighe , G. Kradjian , et al., “The Immunocytokine NHS‐IL12 as a Potential Cancer Therapeutic,” Oncotarget 5, no. 7 (2014): 1869–1884, https://www.ncbi.nlm.nih.gov/pmc/articles/PMC4039112/. 24681847 10.18632/oncotarget.1853 PMC4039112

[24] L. Nadal , F. Peissert , A. Elsayed , et al., “Generation and In Vivo Validation of an IL‐12 Fusion Protein Based on a Novel Anti‐Human FAP Monoclonal Antibody,” Journal for Immunotherapy of Cancer 10, no. 9 (2022): e005282, 10.1136/jitc-2022-005282. 36104101 PMC9476130

[25] E. Gutierrez , M. Bigelow , C. LaCroix , et al., “An Optimized IL‐12‐Fc Expands Its Therapeutic Window, Achieving Strong Activity Against Mouse Tumors at Tolerable Drug Doses,” Med 4, no. 5 (2023): 326–340.e5, 10.1016/j.medj.2023.03.007. 37059099

[26] J. Sharifi , L. A. Khawli , P. Hu , S. King , and A. L. Epstein , “Characterization of a Phage Display‐Derived Human Monoclonal Antibody (NHS76) Counterpart to Chimeric TNT‐1 Directed Against Necrotic Regions of Solid Tumors,” Hybridoma and Hybridomics 20, no. 5–6 (2001): 305–312, 10.1089/15368590152740707. 11839248

[27] Z. Liu and D. Jiao , “Necroptosis, Tumor Necrosis and Tumorigenesis,” Cell Stress 4, no. 1 (2019): 1–8, 10.15698/cst2020.01.208. 31922095 PMC6946014

[28] J. Strauss , J.‐L. Deville , M. Sznol , et al., “First‐In‐Human Phase Ib Trial of M9241 (NHS‐IL12) Plus Avelumab in Patients With Advanced Solid Tumors, Including Dose Expansion in Patients With Advanced Urothelial Carcinoma,” Journal for Immunotherapy of Cancer 11, no. 5 (2023): e005813, 10.1136/jitc-2022-005813. 37236636 PMC10230972

[29] S. Rudman , M. Jameson , M. McKeage , et al., “A Phase 1 Study of AS1409, a Novel Antibody‐Cytokine Fusion Protein, in Patients With Malignant Melanoma or Renal Cell Carcinoma,” Clinical Cancer Research: An Official Journal of the American Association for Cancer Research 17, no. 7 (2011): 1998–2005, 10.1158/1078-0432.CCR-10-2490. 21447719 PMC3071333

[30] A. B. Apolo , J. A. Ellerton , J. R. Infante , et al., “Avelumab as Second‐Line Therapy for Metastatic, Platinum‐Treated Urothelial Carcinoma in the Phase Ib JAVELIN Solid Tumor Study: 2‐Year Updated Efficacy and Safety Analysis,” Journal for Immunotherapy of Cancer 8, no. 2 (2020): e001246, 10.1136/jitc-2020-001246. 33037118 PMC7549450

[31] J. Qian , C. Wang , B. Wang , et al., “The IFN‐γ/PD‐L1 Axis Between T Cells and Tumor Microenvironment: Hints for Glioma Anti‐PD‐1/PD‐L1 Therapy,” Journal of Neuroinflammation 15, no. 1 (2018): 290, 10.1186/s12974-018-1330-2. 30333036 PMC6192101

[32] G. Mariani , A. Lasku , A. Pau , et al., “A Pilot Pharmacokinetic and Immunoscintigraphic Study With the Technetium‐99m‐Labeled Monoclonal Antibody BC‐1 Directed Against Oncofetal Fibronectin in Patients With Brain Tumors,” Cancer 80, no. 12 Suppl (1997): 2484‐2489, 10.1002/(sici)1097-0142(19971215)80:12+<2484::aid-cncr20>3.3.co;2-l. 9406699

[33] K.‐M. Lo , Y. Lan , S. Lauder , et al., “huBC1‐IL12, an Immunocytokine Which Targets EDB‐Containing Oncofetal Fibronectin in Tumors and Tumor Vasculature, Shows Potent Anti‐Tumor Activity in Human Tumor Models,” Cancer Immunology, Immunotherapy 56, no. 4 (2007): 447–457, 10.1007/s00262-006-0203-1. 16874486 PMC11030988

[34] P. Micke and A. Os̈tman , “Tumour‐Stroma Interaction: Cancer‐Associated Fibroblasts as Novel Targets in Anti‐Cancer Therapy?” Lung Cancer 45 (2004): S163–S175, 10.1016/j.lungcan.2004.07.977. 15552797

[35] P. Garin‐Chesa , L. J. Old , and W. J. Rettig , “Cell Surface Glycoprotein of Reactive Stromal Fibroblasts as a Potential Antibody Target in Human Epithelial Cancers,” Proceedings of the National Academy of Sciences of the United States of America 87, no. 18 (1990): 7235–7239, https://www.ncbi.nlm.nih.gov/pmc/articles/PMC54718/. 2402505 10.1073/pnas.87.18.7235 PMC54718

[36] A. A. Fitzgerald and L. M. Weiner , “The Role of Fibroblast Activation Protein in Health and Malignancy,” Cancer Metastasis Reviews 39, no. 3 (2020): 783–803, 10.1007/s10555-020-09909-3. 32601975 PMC7487063

[37] L. Xin , J. Gao , Z. Zheng , et al., “Fibroblast Activation Protein‐α as a Target in the Bench‐To‐Bedside Diagnosis and Treatment of Tumors: A Narrative Review,” Frontiers in Oncology 11 (2021): 648187, 10.3389/fonc.2021.648187. 34490078 PMC8416977

[38] E. Lobner , M. W. Traxlmayr , C. Obinger , and C. Hasenhindl , “Engineered IgG1‐Fc—One Fragment to Bind Them all,” Immunological Reviews 270, no. 1 (2016): 113–131, 10.1111/imr.12385. 26864108 PMC4755133

[39] L. Baudino , Y. Shinohara , F. Nimmerjahn , et al., “Crucial Role of Aspartic Acid at Position 265 in the CH2 Domain for Murine IgG2a and IgG2b Fc‐Associated Effector Functions1,” Journal of Immunology 181, no. 9 (2008): 6664–6669, 10.4049/jimmunol.181.9.6664. 18941257

[40] E. F. Zhu , S. A. Gai , C. F. Opel , et al., “Synergistic Innate and Adaptive Immune Response to Combination Immunotherapy With Anti‐Tumor Antigen Antibodies and Extended Serum Half‐Life IL‐2,” Cancer Cell 27, no. 4 (2015): 489–501, 10.1016/j.ccell.2015.03.004. 25873172 PMC4398916

[41] S. I. Kim , C. R. Cassella , and K. T. Byrne , “Tumor Burden and Immunotherapy: Impact on Immune Infiltration and Therapeutic Outcomes,” Frontiers in Immunology 11 (2021): 629722, 10.3389/fimmu.2020.629722. 33597954 PMC7882695

[42] C. Gialeli , A. D. Theocharis , and N. K. Karamanos , “Roles of Matrix Metalloproteinases in Cancer Progression and Their Pharmacological Targeting,” FEBS Journal 278, no. 1 (2011): 16–27, 10.1111/j.1742-4658.2010.07919.x. 21087457

[43] D. H. Presky , L. J. Minetti , S. Gillessen , et al., “Analysis of the Multiple Interactions Between IL‐12 and the High Affinity IL‐12 Receptor Complex,” Journal of Immunology 160, no. 5 (1998): 2174–2179, 10.4049/jimmunol.160.5.2174. 9498755

[44] M. Fabbri , C. Smart , and R. Pardi , “T Lymphocytes,” The International Journal of Biochemistry & Cell Biology 35, no. 7 (2003): 1004–1008, 10.1016/S1357-2725(03)00037-2. 12672468

[45] M. W. Rohaan , S. Wilgenhof , and J. B. A. G. Haanen , “Adoptive Cellular Therapies: The Current Landscape,” Virchows Archiv 474, no. 4 (2019): 449–461, 10.1007/s00428-018-2484-0. 30470934 PMC6447513

[46] S. A. Rosenberg , J. R. Yannelli , J. C. Yang , et al., “Treatment of Patients With Metastatic Melanoma With Autologous Tumor‐Infiltrating Lymphocytes and Interleukin 2,” JNCI Journal of the National Cancer Institute 86, no. 15 (1994): 1159–1166, 10.1093/jnci/86.15.1159. 8028037

[47] Y. Zhao , J. Deng , S. Rao , et al., “Tumor Infiltrating Lymphocyte (TIL) Therapy for Solid Tumor Treatment: Progressions and Challenges,” Cancers 14, no. 17 (2022): 4160, 10.3390/cancers14174160. 36077696 PMC9455018

[48] S. T. Paijens , A. Vledder , M. de Bruyn , and H. W. Nijman , “Tumor‐Infiltrating Lymphocytes in the Immunotherapy Era,” Cellular and Molecular Immunology 18, no. 4 (2021): 842–859, 10.1038/s41423-020-00565-9. 33139907 PMC8115290

[49] C. Yee , J. A. Thompson , D. Byrd , et al., “Adoptive T Cell Therapy Using Antigen‐Specific CD8+ T Cell Clones for the Treatment of Patients With Metastatic Melanoma: In Vivo Persistence, Migration, and Antitumor Effect of Transferred T Cells,” Proceedings of the National Academy of Sciences of the United States of America 99, no. 25 (2002): 16168–16173, 10.1073/pnas.242600099. 12427970 PMC138583

[50] A. P. Rapoport , E. A. Stadtmauer , G. K. Binder‐Scholl , et al., “NY‐ESO‐1–Specific TCR–Engineered T Cells Mediate Sustained Antigen‐Specific Antitumor Effects in Myeloma,” Nature Medicine 21, no. 8 (2015): 914–921, 10.1038/nm.3910. PMC4529359 26193344

[51] P. F. Robbins , R. A. Morgan , S. A. Feldman , et al., “Tumor Regression in Patients With Metastatic Synovial Cell Sarcoma and Melanoma Using Genetically Engineered Lymphocytes Reactive With NY‐ESO‐1,” Journal of Clinical Oncology 29, no. 7 (2011): 917–924, 10.1200/JCO.2010.32.2537. 21282551 PMC3068063

[52] C. Szeto , C. A. Lobos , A. T. Nguyen , and S. Gras , “TCR Recognition of Peptide–MHC‐I: Rule Makers and Breakers,” International Journal of Molecular Sciences 22, no. 1 (2021): 68, 10.3390/ijms22010068. PMC7793522 33374673

[53] N. Aptsiauri , F. Ruiz‐Cabello , and F. Garrido , “The Transition From HLA‐I Positive to HLA‐I Negative Primary Tumors: The Road to Escape From T‐Cell Responses,” Current Opinion in Immunology 51 (2018): 123–132, 10.1016/j.coi.2018.03.006. 29567511

[54] S. Feins , W. Kong , E. F. Williams , M. C. Milone , and J. A. Fraietta , “An Introduction to Chimeric Antigen Receptor (CAR) T‐Cell Immunotherapy for Human Cancer,” American Journal of Hematology 94, no. S1 (2019): S3–S9, 10.1002/ajh.25418. 30680780

[55] S. A. Grupp , M. Kalos , D. Barrett , et al., “Chimeric Antigen Receptor‐Modified T Cells for Acute Lymphoid Leukemia,” New England Journal of Medicine 368, no. 16 (2013): 1509–1518, 10.1056/NEJMoa1215134. 23527958 PMC4058440

[56] B. G. Till , M. C. Jensen , J. Wang , et al., “Adoptive Immunotherapy for Indolent Non‐Hodgkin Lymphoma and Mantle Cell Lymphoma Using Genetically Modified Autologous CD20‐Specific T Cells,” Blood 112, no. 6 (2008): 2261–2271, 10.1182/blood-2007-12-128843. 18509084 PMC2532803

[57] S. A. Rosenberg , B. S. Packard , P. M. Aebersold , et al., “Use of Tumor‐Infiltrating Lymphocytes and Interleukin‐2 in the Immunotherapy of Patients With Metastatic Melanoma. A Preliminary Report,” New England Journal of Medicine 319, no. 25 (1988): 1676–1680, 10.1056/NEJM198812223192527. 3264384

[58] L. Ginaldi , M. De Martinis , E. Matutes , N. Farahat , R. Morilla , and D. Catovsky , “Levels of Expression of CD19 and CD20 in Chronic B Cell Leukaemias,” Journal of Clinical Pathology 51, no. 5 (1998): 364–369 https://www.ncbi.nlm.nih.gov/pmc/articles/PMC500695/. 9708202 10.1136/jcp.51.5.364 PMC500695

[59] Z. Fu , J. Zhou , R. Chen , et al., “Cluster of Differentiation 19 Chimeric Antigen Receptor T‐Cell Therapy in Pediatric Acute Lymphoblastic Leukemia,” Oncology Letters 20, no. 4 (2020): 36, 10.3892/ol.2020.11897. 32802160 PMC7412636

[60] M. Martinez and E. K. Moon , “CAR T Cells for Solid Tumors: New Strategies for Finding, Infiltrating, and Surviving in the Tumor Microenvironment,” Frontiers in Immunology 10 (2019): 128, 10.3389/fimmu.2019.00128. 30804938 PMC6370640

[61] C. J. Henry , D. A. Ornelles , L. M. Mitchell , K. L. Brzoza‐Lewis , and E. M. Hiltbold , “IL‐12 Produced by Dendritic Cells Augments CD8+ T Cell Activation Through the Production of the Chemokines CCL1 and CCL17,” Journal of Immunology (Baltimore, Md.: 1950) 181, no. 12 (2008): 8576–8584, https://www.ncbi.nlm.nih.gov/pmc/articles/PMC2716729/. 19050277 10.4049/jimmunol.181.12.8576 PMC2716729

[62] G. R. Starbeck‐Miller , H.‐H. Xue , and J. T. Harty , “IL‐12 and Type I Interferon Prolong the Division of Activated CD8 T Cells by Maintaining High‐Affinity IL‐2 Signaling in Vivo,” Journal of Experimental Medicine 211, no. 1 (2013): 105–120, 10.1084/jem.20130901. 24367005 PMC3892973

[63] J. Valenzuela , C. Schmidt , and M. Mescher , “The Roles of IL‐12 in Providing a Third Signal for Clonal Expansion of Naive CD8 T Cells1,” Journal of Immunology 169, no. 12 (2002): 6842–6849, 10.4049/jimmunol.169.12.6842. 12471116

[64] S. P. Kerkar , P. Muranski , A. Kaiser , et al., “Tumor‐Specific CD8+ T Cells Expressing Interleukin‐12 Eradicate Established Cancers in Lymphodepleted Hosts,” Cancer Research 70, no. 17 (2010): 6725–6734, 10.1158/0008-5472.CAN-10-0735. 20647327 PMC2935308

[65] D. Chinnasamy , Z. Yu , S. P. Kerkar , et al., “Local Delivery of Interleukin‐12 Using T Cells Targeting VEGF Receptor‐2 Eradicates Multiple Vascularized Tumors in Mice,” Clinical Cancer Research: An Official Journal of the American Association for Cancer Research 18, no. 6 (2012): 1672–1683, 10.1158/1078-0432.CCR-11-3050. 22291136 PMC6390958

[66] M.‐G. Pan , Y. Xiong , and F. Chen , “NFAT Gene Family in Inflammation and Cancer,” Current Molecular Medicine 13, no. 4 (2013): 543–554, https://www.ncbi.nlm.nih.gov/pmc/articles/PMC3694398/. 22950383 10.2174/1566524011313040007 PMC3694398

[67] Y. Yang , H. Yang , Y. Alcaina , et al., “Inducible Expression of Interleukin‐12 Augments the Efficacy of Affinity‐Tuned Chimeric Antigen Receptors in Murine Solid Tumor Models,” Nature Communications 14, no. 1 (2023): 2068, 10.1038/s41467-023-37646-y. PMC10097865 37045815

[68] L. Zhang , R. A. Morgan , J. Beane , et al., “Tumor Infiltrating Lymphocytes Genetically Engineered With an Inducible Gene Encoding Interleukin‐12 for the Immunotherapy of Metastatic Melanoma,” Clinical Cancer Research: An Official Journal of the American Association for Cancer Research 21, no. 10 (2015): 2278–2288, 10.1158/1078-0432.CCR-14-2085. 25695689 PMC4433819

[69] D. Campillo‐Davo , M. De Laere , G. Roex , et al., “The Ins and Outs of Messenger RNA Electroporation for Physical Gene Delivery in Immune Cell‐Based Therapy,” Pharmaceutics 13, no. 3 (2021): 396, 10.3390/pharmaceutics13030396. 33809779 PMC8002253

[70] C. J. Turtle , L.‐A. Hanafi , C. Berger , et al., “Immunotherapy of Non‐Hodgkin Lymphoma With a Defined Ratio of CD8+ and CD4+ CD19‐Specific Chimeric Antigen Receptor‐Modified T Cells,” Science Translational Medicine 8, no. 355 (2016): 355ra116, 10.1126/scitranslmed.aaf8621. PMC5045301 27605551

[71] H. Meister , T. Look , P. Roth , et al., “Multifunctional mRNA‐Based CAR T Cells Display Promising Antitumor Activity Against Glioblastoma,” Clinical Cancer Research 28, no. 21 (2022): 4747–4756, 10.1158/1078-0432.CCR-21-4384. 36037304

[72] C. A. Di Trani , A. Cirella , L. Arrizabalaga , et al., “Intracavitary Adoptive Transfer of IL‐12 mRNA‐Engineered Tumor‐Specific CD8+ T Cells Eradicates Peritoneal Metastases in Mouse Models,” Oncoimmunology 12, no. 1 (2023): 2147317, 10.1080/2162402X.2022.2147317. 36531687 PMC9757485

[73] M. A. Aznar , N. Tinari , A. J. Rullán , A. R. Sánchez‐Paulete , M. E. Rodriguez‐Ruiz , and I. Melero , “Intratumoral Delivery of Immunotherapy—Act Locally, Think Globally,” Journal of Immunology 198, no. 1 (2017): 31‐39, 10.4049/jimmunol.1601145. 27994166

[74] W. X. Hong , S. Haebe , A. S. Lee , et al., “Intratumoral Immunotherapy for Early‐Stage Solid Tumors,” Clinical Cancer Research 26, no. 13 (2020): 3091–3099, 10.1158/1078-0432.CCR-19-3642. 32071116 PMC7439755

[75] E. H. J. Lee , J. P. Murad , L. Christian , et al., “Antigen‐Dependent IL‐12 Signaling in CAR T Cells Promotes Regional to Systemic Disease Targeting,” Nature Communications 14 (2023): 4737, 10.1038/s41467-023-40115-1. PMC10406808 37550294

[76] Y. Luo , Z. Chen , M. Sun , et al., “IL‐12 Nanochaperone‐Engineered CAR T Cell for Robust Tumor‐Immunotherapy,” Biomaterials 281 (2022): 121341, 10.1016/j.biomaterials.2021.121341. 34995901

[77] F. Arabi , V. Mansouri , and N. Ahmadbeigi , “Gene Therapy Clinical Trials, Where Do We go? An Overview,” Biomedicine & Pharmacotherapy 153 (2022): 113324, 10.1016/j.biopha.2022.113324. 35779421

[78] L. Xie , Y. Han , Y. Liu , et al., “Viral Vector‐Based Cancer Treatment and Current Clinical Applications,” MedComm—Oncology 2, no. 4 (2023): e55, 10.1002/mog2.55.

[79] C. Tang , L. Li , T. Mo , et al., “Oncolytic Viral Vectors in the Era of Diversified Cancer Therapy: From Preclinical to Clinical,” Clinical & Translational Oncology 24, no. 9 (2022): 1682–1701, 10.1007/s12094-022-02830-x. 35612653 PMC9131313

[80] A. A. Alkayyal , A. B. Mahmoud , and R. C. Auer , “Interleukin‐12‐Expressing Oncolytic Virus: A Promising Strategy for Cancer Immunotherapy,” Journal of Taibah University Medical Sciences 11, no. 3 (2016): 187–193, 10.1016/j.jtumed.2016.04.002.

[81] A. Cirella , C. Luri‐Rey , C. A. Di Trani , et al., “Novel Strategies Exploiting Interleukin‐12 in Cancer Immunotherapy,” Pharmacology & Therapeutics 239 (2022): 108189, 10.1016/j.pharmthera.2022.108189. 35430292

[82] K. G. Nguyen , M. R. Vrabel , S. M. Mantooth , et al., “Localized Interleukin‐12 for Cancer Immunotherapy,” Frontiers in Immunology 11 (2020): 575597, 10.3389/fimmu.2020.575597. 33178203 PMC7593768

[83] B. Sangro , G. Mazzolini , J. Ruiz , et al., “Phase I Trial of Intratumoral Injection of an Adenovirus Encoding Interleukin‐12 for Advanced Digestive Tumors,” Journal of Clinical Oncology 22, no. 8 (2004): 1389–1397, 10.1200/JCO.2004.04.059. 15084613

[84] D. Estevez‐Ordonez , J. Stein , P. Maleknia , et al., “CTIM‐13. Phase I Clinical Trial of Oncolytic HSV‐1 M032, A Second‐Generation Virus Armed to Expressed Il‐12, for the Treatment of Adult Patients With Recurrent or Progressive Malignant Glioma,” Neuro‐Oncology 25, no. Supplement_5 (2023): 64, 10.1093/neuonc/noad179.0253.

[85] E. A. Chiocca , J. S. Yu , R. V. Lukas , et al., “Regulatable Interleukin‐12 Gene Therapy in Patients With Recurrent High‐Grade Glioma: Results of a Phase 1 Trial,” Science Translational Medicine 11, no. 505 (2019): eaaw5680, 10.1126/scitranslmed.aaw5680. 31413142 PMC7286430

[86] M. P. Velders , S. McElhiney , M. C. Cassetti , et al., “Eradication of Established Tumors by Vaccination With Venezuelan Equine Encephalitis Virus Replicon Particles Delivering Human Papillomavirus 16 E7 RNA1,” Cancer Research 61, no. 21 (2001): 7861–7867. 11691804

[87] R. Yamanaka , S. A. Zullo , J. Ramsey , et al., “Induction of Therapeutic Antitumor Antiangiogenesis by Intratumoral Injection of Genetically Engineered Endostatin‐Producing Semliki Forest Virus,” Cancer Gene Therapy 8, no. 10 (2001): 796–802, 10.1038/sj.cgt.7700367. 11687903

[88] K. Lundstrom , “Alphaviruses in Immunotherapy and Anticancer Therapy,” Biomedicine 10, no. 9 (2022): 2263, 10.3390/biomedicines10092263. PMC9496634 36140364

[89] F. P. Roche , B. J. Sheahan , S. M. O'Mara , and G. J. Atkins , “Semliki Forest Virus‐Mediated Gene Therapy of the RG2 Rat Glioma,” Neuropathology and Applied Neurobiology 36, no. 7 (2010): 648–660, 10.1111/j.1365-2990.2010.01110.x. 20649937

[90] S. Opp , A. Hurtado , C. Pampeno , Z. Lin , and D. Meruelo , “Potent and Targeted Sindbis Virus Platform for Immunotherapy of Ovarian Cancer,” Cells 12, no. 1 (2022): 77, 10.3390/cells12010077. 36611875 PMC9818975

[91] K. Ye , F. Li , R. Wang , et al., “An Armed Oncolytic Virus Enhances the Efficacy of Tumor‐Infiltrating Lymphocyte Therapy by Converting Tumors to Artificial Antigen‐Presenting Cells in Situ,” Molecular Therapy 30, no. 12 (2022): 3658–3676, 10.1016/j.ymthe.2022.06.010. 35715953 PMC9734027

[92] H.‐M. Nguyen and D. Saha , “The Current State of Oncolytic Herpes Simplex Virus for Glioblastoma Treatment,” Oncolytic Virotherapy 10 (2021): 1–27, 10.2147/OV.S268426. 33659221 PMC7917312

[93] J. N. Parker , G. Y. Gillespie , C. E. Love , S. Randall , R. J. Whitley , and J. M. Markert , “Engineered Herpes Simplex Virus Expressing IL‐12 in the Treatment of Experimental Murine Brain Tumors,” Proceedings of the National Academy of Sciences 97, no. 5 (2000): 2208–2213, 10.1073/pnas.040557897. PMC15779 10681459

[94] C. H. Quinn , A. M. Beierle , S. C. Hutchins , et al., “Targeting High‐Risk Neuroblastoma Patient‐Derived Xenografts With Oncolytic Virotherapy,” Cancers 14, no. 3 (2022): 762, 10.3390/cancers14030762. 35159029 PMC8834037

[95] E. T. Wong , K. R. Hess , M. J. Gleason , et al., “Outcomes and Prognostic Factors in Recurrent Glioma Patients Enrolled Onto Phase II Clinical Trials,” Journal of Clinical Oncology: Official Journal of the American Society of Clinical Oncology 17, no. 8 (1999): 2572–2578, 10.1200/JCO.1999.17.8.2572. 10561324

[96] F. Zemp , J. Rajwani , and D. J. Mahoney , “Rhabdoviruses as Vaccine Platforms for Infectious Disease and Cancer,” Biotechnology and Genetic Engineering Reviews 34, no. 1 (2018): 122–138, 10.1080/02648725.2018.1474320. 29781359

[97] F. Tzelepis , H. K. Birdi , A. Jirovec , et al., “Oncolytic Rhabdovirus Vaccine Boosts Chimeric Anti‐DEC205 Priming for Effective Cancer Immunotherapy,” Molecular Therapy ‐ Oncolytics 19 (2020): 240–252, 10.1016/j.omto.2020.10.007. 33209979 PMC7658579

[98] Y. Zhang and B. M. Nagalo , “Immunovirotherapy Based on Recombinant Vesicular Stomatitis Virus: Where Are We?” Frontiers in Immunology 13 (2022): 898631, 10.3389/fimmu.2022.898631. 35837384 PMC9273848

[99] R. H. Abdulal , J. S. Malki , E. Ghazal , et al., “Construction of VSVΔ51M Oncolytic Virus Expressing Human Interleukin‐12,” Frontiers in Molecular Biosciences 10 (2023): 1190669, 10.3389/fmolb.2023.1190669. 37255540 PMC10225647

[100] J. A. Barrett , H. Cai , J. Miao , et al., “Regulated Intratumoral Expression of IL‐12 Using a RheoSwitch Therapeutic System® (RTS®) Gene Switch as Gene Therapy for the Treatment of Glioma,” Cancer Gene Therapy 25, no. 5 (2018): 106–116, 10.1038/s41417-018-0019-0. 29755109 PMC6021367

[101] S. K. Watkins , N. K. Egilmez , J. Suttles , and R. D. Stout , “IL‐12 Rapidly Alters the Functional Profile of Tumor‐Associated and Tumor‐Infiltrating Macrophages In Vitro and In Vivo,” Journal of Immunology 178, no. 3 (2007): 1357–1362, 10.4049/jimmunol.178.3.1357. 17237382

[102] A. M. Newman , C. B. Steen , C. L. Liu , et al., “Determining Cell Type Abundance and Expression From Bulk Tissues With Digital Cytometry,” Nature Biotechnology 37, no. 7 (2019): 773–782, 10.1038/s41587-019-0114-2. PMC6610714 31061481

[103] Yu, X.‐L. , Wu, B.‐T. , Ma, T.‐T. , Lin, Y. , Cheng, F. , Xiong, H.‐Y. , Xie, C.‐L. , Liu, C.‐Y. , Wang, Q. , Li, Z.‐W. , & Tu, Z.‐G. (2016). Overexpression of IL‐12 Reverses the Phenotype and Function of M2 Macrophages to M1 Macrophages.

[104] Y. Togashi , K. Shitara , and H. Nishikawa , “Regulatory T Cells in Cancer Immunosuppression—Implications for Anticancer Therapy,” Nature Reviews Clinical Oncology 16, no. 6 (2019): 356–371, 10.1038/s41571-019-0175-7. 30705439

[105] B. Mirlekar and Y. Pylayeva‐Gupta , “IL‐12 Family Cytokines in Cancer and Immunotherapy,” Cancers 13, no. 2 (2021): 167, 10.3390/cancers13020167. 33418929 PMC7825035

[106] Y. Hong , Y. Robbins , X. Yang , et al., “Cure of Syngeneic Carcinomas With Targeted IL‐12 Through Obligate Reprogramming of Lymphoid and Myeloid Immunity,” JCI Insight 7, no. 5 (2020): e157448, 10.1172/jci.insight.157448. PMC8983130 35260537

[107] P. Kornprat , M. J. Pollheimer , R. A. Lindtner , A. Schlemmer , P. Rehak , and C. Langner , “Value of Tumor Size as a Prognostic Variable in Colorectal Cancer: A Critical Reappraisal,” American Journal of Clinical Oncology 34, no. 1 (2011): 43–49, 10.1097/COC.0b013e3181cae8dd. 20101166

[108] K. Markou , J. Goudakos , S. Triaridis , J. Konstantinidis , V. Vital , and A. Nikolaou , “The Role of Tumor Size and patient's age as Prognostic Factors in Laryngeal Cancer,” Hippokratia 15, no. 1 (2011): 75–80. 21607041 PMC3093151

